# Age, ageing, ageism and “age-itation” in the Age of COVID-19: rights and obligations relating to older persons in Israel as observed through the lens of medical ethics

**DOI:** 10.1186/s13584-020-00416-y

**Published:** 2020-11-12

**Authors:** A. Mark Clarfield, Alan Jotkowitz

**Affiliations:** 1 Geriatric Medicine, Centre for Global Health and the Medical School for International Health, Faculty of Health Sciences, Ben-Gurion University of the Negev, MSIH-Bet Caroline, PO Box 653, 8410501 Beer-sheva, Israel; 2 McGill University, Montreal, Canada; 3 Medical School for International Health and The Jakobovits Center for Jewish Medical Ethics, Faculty of Health Sciences, Ben-Gurion University of the Negev, Beer-sheva, Israel

## Abstract

COVID-19, the illness caused by the SARS-CoV-2 virus, has reached pandemic proportions. Although the virus can cause disease in anyone, it is particularly dangerous for those with various “co-morbidities” such as heart disease, hypertension, diabetes, obesity and others. Furthermore, advancing age (from about 60 on), even in those older persons without any accompanying illnesses, is a strong and independent risk factor for pneumonia, need for an ICU bed and death from the virus. It is therefore essential to find ways to protect all at-risk persons (old or young) from the virus but at the same time not harming, more than absolutely necessary their essential freedoms as well as taking into account their social/psychological needs. Compared with other OECD countries, Israel’s population is still relatively young, with only 11.5% being over 65+ with a smaller proportion of older persons in long-term institutions than that found in most other comparable jurisdictions. These factors might explain a part of the country’s (so far) relatively low rates of serious disease and mortality compared to those seen in other developed countries. However there are still over a million older citizens at risk and the numbers of infected, hospitalized and seriously ill persons are rising once again. This is no time for complacency.

An analysis of the effect of age on the disease as seen through the principles of medical ethics is followed by a proposal as to how best to balance these sometimes conflicting goals. This paper relates mainly to older persons in the community since the Ministry of Health early on in the pandemic initiated an effective program (Magen Avot) meant to protect those older persons in long-term care institutions. Recommendations include the Ministry of Health publishing clear guidelines as to risk factors and offering sensible advice on how to practice physical (not “social”) distancing without exacerbating an older person’s sense of social isolation. In order to reduce the incidence of influenza (which can clinically be confused with COVID-19) and the potentially disastrous consequences of a “double pandemic” this coming winter, a robust flu vaccination program needs immediate implementation. Persons at all ages (but especially those 60+) should be encouraged and assisted to sign advance directives, especially those who do not wish to undergo invasive therapy. An individual older person’s wish to “make way” for younger people should be respected as an expression of his/her autonomy. As we enter the second wave, triage mechanisms and protocols need to be circulated in readiness for and well before a situation in which an acute imbalance develops between the availability for acute resources and the population’s need for them. The Ministry of Health, in cooperation with other relevant ministries and NGOs, should take the lead in developing plans, ensuring that they are carried out in an orderly, timely and transparent manner. The blanket is indeed not large enough but we must place it as judiciously as possible in order as much as possible to protect, cover and keep warm the body politic.

**Supplementary information:**

**Supplementary information** accompanies this paper at 10.1186/s13584-020-00416-y.

## Introduction

The SARS-CoV-2 virus can effect anyone at any age. As it continues to spread throughout the world it will clearly be with us for the foreseeable future. Fortunately, at any age, almost all infected people (even older persons) will overcome this infection without serious sequelae. That being said, the waning immunological vigour of older persons and presence of risk factors (“co-morbidity”) often accompanying older age (hypertension, increased BMI, diabetes, chronic lung disease, immunomodulation-immunosuppression, smoking, ischemic heart disease and cerebrovascular disease, etc.) make the disease a much more serious event for many older people. Furthermore, it presents special challenges to their doctors and to the health care system (ref [1]).

For those infected, the incidence of severe disease rises inexorably and logarithmically with chronological age (beginning around age 60). Comorbidity adds to the risk but does not replace age as an independent factor. Some older patients will develop viral pneumonia and a relatively small subgroup will require ICU and ventilator support. However, should these numbers grow too quickly, they can easily overwhelm the number of medical staff, hospital beds and ventilators available - a dire situation already observed in several places around the world. Many of these acutely ill patients will die and the majority, even if they do survive, remain in the ICU for many weeks.

In this paper we address the issue of old age and how this particular coronavirus specifically effects older people with Israel’s experience being the framework for this exploration. In order to do so we first describe the country’s demography briefly followed by a few words about formal Jewish law (Halacha) and its role (or surprising lack thereof) in framing health policy. Next, the issue of aging itself is dissected: its role as a risk factor for and the influence of lockdown policies on older persons. As well we define “ageism”, explaining the concept’s relevance: what it is and what it is not as well as how ageism can adversely affect older persons during this crisis.

We then analyze some of the trenchant dilemmas relating to COVID-19 through the lens of medical ethics, examining the issues of triage and distributive justice, maximizing non-maleficence and how to ensure that older people’s autonomy is facilitated while ensuring that they also remain safe (beneficence). Given all of the aforementioned, we offer a specific program for how to move forward and help older persons and the health system to stay afloat as we try to ride the second wave of the pandemic without drowning.

### The new virus in Israel: a young-old country

Compared with most other industrialized countries, Israel’s population is still relatively young. This demographic pattern effects the expression of the pandemic in the country. See sidebar 1 for details.

### Israel in the world

Across the globe the reported case-fatality rate (ratio between confirmed deaths and confirmed cases) for COVID-19 is around 2.8% (more than 10 times that for influenza), but this number can vary widely by location - as high as 9.7% (Italy) to less than 0.3% (Iceland) (https://ourworldindata.org/grapher/coronavirus-cfr; accessed 17 Oct., 2020). In Israel, at least so far (as of late October, 2020), this rate is still relatively low at 0.7% and despite the very sharp recent rise in confirmed cases it has held reasonably steady over the past few months. Examining another ratio (again, as of late October, 2020), 231 per million population have died here compared with 637 in the UK or 672 in the US, although the rate in Israel may rise given the recent increase in new cases observed. (See https://www.worldometers.info/coronavirus/ accessed October 17, 2020).

For various reasons, early on, in the spring of 2020, the country coped quite well - with a very low infection rate, few severe cases and a very low death rate. However, coinciding with (or because of) a too rapid release from the lockdown, the figures have deteriorated over the past few months, especially recently (late October). For example, according to the Ministry of Health’s (Hebrew language) dashboard (see https://datadashboard.health.gov.il/COVID-19/?utm_source=go.gov.il&utm_medium=referral; accessed 17 Oct., 2020) the number of new confirmed cases almost doubled in the last week of August 2020 and the number of severe *and* ventilated cases has gone up by 20% in the last week of August. As of late September the government had reclosed the country more or less hermetically after a failed policy of quarantining “hot” cities or neighbourhoods with particularly high rates of infection - most of these being Haredi (ultra–orthodox) or Arab municipalities.

As such, beginning just before Judaism’s most holy Day of Atonement (*Yom Kippur*) the country has once again been hermetically closed down for a planned two week (at least) period. This after the failure of localized urban quarantines which failed to dampen the epidemic - most of these being in Haredi (ultra–orthodox) or Arab municipalities. This picture can hardly be considered successful policy management even if there remains legitimate argument about the variance caused by each of the many steps taken (or not) to date. Clearly, these numbers express a moving target and it is not easy nor often possible to tease out the exact cause and effect for rising or falling rates which may be affected by many variables such as change in or extent of testing policy and others. Furthermore, improved treatment protocols have brought the pressure for ICU/ventilator resources down somewhat. Equally important, with respect to a possible exhaustion of the health system through care seeking, the number of hospital beds, trained personnel etc. may be much more important than the number of ventilators.

Overall, given the larger number of asymptomatic people than those identified as infected, the overall death rate for those actually infected may be even lower than that reported. However, we do not yet know if all those who do survive serious illness will return to their premorbid state of health and function. There is some doubt that this will be the case, with a prediction that a significant minority may suffer serious long-term sequelae subsequently requiring rehabilitation (ref [2]).

In Israel, in response to this pandemic, as has been the case in many countries, initially broad and very strict social distancing measures were enacted for the whole population. However, after the number of cases fell precipitously in May these strictures were loosened. Unfortunately, despite warnings by relevant professionals, this release was allowed to take place much too quickly and in a rather haphazard manner, with a resultant recrudescence of cases.

Not surprisingly, many older people found these lockdown steps very difficult to tolerate as a result of being almost totally cut off from family and friends, not to speak of having to look after themselves with minimal help from outside. There is concern that the dangers of social isolation for older people may equal or even outweigh its benefits (ref [3]). As such, some have called for a useful change in terminology from “social” to “physical” distancing. Furthermore, some older people in Israel were skeptical of the government’s motives and felt that they were being “sacrificed” to keep the medical system functioning in order to favour younger citizens. For their part, many younger people continue to express skepticism relating to the dangers of SARS-CoV-2 as a result of their low chance of suffering a complication should they become infected and in part because of their understandable lack of faith in the present government and its actions.

### Peering ahead

Although we are dealing with a fast and erratically moving target, with the present situation in mind this paper will elucidate relevant issues and offer policy recommendations germane to when and how older persons can minimize risk and at some point in the future return to their pre-COVID-19 routine in Israel. The general approach taken is that of a “soft utilitarianism” (i.e. what promises the greatest good to the greatest number) while at the same time we make every effort to minimize damage to individual human rights and to ensure that the scourge of ageism does not manifest itself.

Against the odds, if the epidemic once again quickly subsides, the issues addressed herein will be much less relevant. However, this paper is meant to deal with the much more probable scenario in which SARS-CoV-2 will be with us for months, perhaps years to come, and especially as we now suffer a second wave more severe than the first. And as was the case in the 1919/20 influenza epidemic, we may even have to endure a third wave.

Around the world, as a first and necessary step, blanket physical distancing has proven itself, as it did in previous influenza pandemics (both 1918 and 2009). Along with heightened personal hygiene, hand washing, and especially the use of face masks, this blunt instrument had until very recently (mid-June) largely reduced and delayed peak attack rates in many countries as well as reducing mortality and the number of very sick persons requiring hospital care (ref [4]). In Israel and elsewhere, but unfortunately not everywhere (see northern Italy ref [5] and Spain), this tool helped save many lives as well as reducing pressure on acute and ICU hospital beds, of which Israel is lacking. As well, this step has helped at least so far to preserve precious ICU/ventilator beds for the use of young and old alike.

Along with the whole population so far, at least physically, older persons have benefitted from these drastic measures, although the national economy is taking a severe blow adding not surprisingly to social and political instability. As the country locks down again, the question arises once more as to what approach should be taken. An excellent overview of how to manage such a challenge can be found in Tomas Pueyo’s much cited article “The Hammer and the Dance” in which the “hammer” refers to the lockdown resulting in abrupt social distancing meant to flatten the curve and the “dance” to how we can get out of lockdown with the least possible loss of life whilst making every effort to maintain the economy (https://medium.com/@tomaspueyo/coronavirus-the-hammer-and-the-dance-be9337092b56).

Explaining the hammer in an interview Pueyo stated, “I wanted to create a very strong metaphor ….that could represent the idea of something aggressive early on and then something less aggressive afterwards.” He termed the next phase a dance …because it is a much more fluid phase. You need to know the steps of the dance and really apply them as if it were choreography. (See: https://abc7news.com/society/viral-hammer-and-the-dance-influences-reopening-amid-pandemic/6199923/ accessed 3 Sept., 2020).

With respect to older persons, however much it reduces risk, there is justifiable concern over the real health costs involved in physical distancing by keeping older persons confined too strictly and for too long to their homes. These include ill effects, both medical and psychological, especially on those of low socio-economic status (https://www.nytimes.com/2020/06/08/opinion/coronavirus-elderly-suicide.html). Furthermore, there is some early anecdotal evidence from both here and abroad that many people have delayed clinic or ER visits for non-coronavirus conditions, putting their overall health at risk. It is also not clear under lockdown how many isolated older persons have been able to manage their day to day affairs – groceries, medications, household cleaning and repairs. All this is especially problematic when these people cannot avail themselves of the help of their children and/or neighbours. Without this aid it is difficult for such older persons to cope with social isolation and resulting loneliness.

In order to analyze this complex issue, whilst taking a morally defensible ethical stance, the approach herein attempts to balance the sometimes conflicting principles of medical ethics, namely: *autonomy, beneficence (*doing good*), non –maleficence (*not doing evil*) and distributive justice.*

### The approach of formal Jewish law (Halacha)

With 80% of its population being Jewish and given the country’s unique history, it will come as no surprise that Jewish law and traditions will sometimes influence both Israel’s norms and laws. (See sidebar 2.)

### Age, ageing, and ageism

#### Biological ageing: what is it?

The phenomenon of ageing does not necessarily lead to disease but it does gradually reduce the human organism’s ability to withstand stress and is thus relevant to considerations re the effects of the SARS-CoV-2 virus on older persons. (See sidebar 3).

#### Age as a risk factor

Just as for many other diseases, there are “risk factors” for developing COVID-19, this term refers not to the disease per se but as something that increases a person’s chances of developing one. For example, cigarette smoking is a risk factor for lung cancer, as is the metabolic syndrome for heart disease. However, having a risk factor does not guarantee that one will inevitably develop the illness in question. It just makes the disease more likely. For its part, chronological age (even when controlling for other characteristics) is clearly one of the most significant risk factors for COVID-19 pneumonia, the need for ventilator support and above all for death (ref [6]). Why this is and what implications this fact might have for relevant policies will now be addressed.

#### The SARS-CoV-2 virus and its correlations with age and ageing

Fortunately, for reasons not yet clear, young people (especially children 1–9 years old) seem hardly to be affected by this coronavirus. Although they are indeed very efficient spreaders to adults for influenza, it appears that with the coronavirus this may fortunately *not* be the case. Furthermore, although further work needs to be done to reach a firm conclusion, it is possible that young children may actually not constitute a significant danger to their teachers, parents or grandparents.

However, at the other end of the spectrum, as alluded to above, increasing age *is* most definitely an independent risk factor for complications and death once a person is infected (ref [6]). For example, an intensive care audit from the UK showed a very poor COVID-19 pneumonia ICU survival rate for those over 70 of less than a quarter (only 24.3%) versus more than three quarters survival (75.7%) for those 16–49 years of age (ref [7]). A more recent study from northern Italy indicated an equally dire prognosis for older men admitted to ICU with a death rate of 40% for those 71–80 years old and 52% for those 80–90 (ref [8]). The numbers were even higher if the patient had hypertension. There are similar figures from Israeli ICUs and elsewhere across the world. Tragically, although treatment protocols have indeed increased the chance of survival at all ages, the bottom line is that older persons who become ill enough to require ventilator support are very unlikely to survive.

An understandable point has been made that not all older persons are the same. For example, it has been argued that one can find an 85 year old who by a combination of good fortune, favourable genetics and careful lifestyle choices, is in better health than an individual 75 year old with none of these three characteristics. In other words, the “biological” age of a particular 85 year old may well be less than the chronological age of an individual younger by a decade or even two. While this may occasionally be the case, it would be very difficult to assess this phenomenon in any accurate or scientific way *within* an age cohort (e.g. 65–80 or 80+).

And unfortunately, despite the wishful thinking of many older persons and some mistaken authorities, the facts show that the older one is, the higher the risk even when controlling for various relevant co-morbidities. One study indicated that an 80 + year old with no known diseases still has fewer years left to live than does an 60–69 year old with 5 (!) co-morbidities (ref [9]). Sadly, these facts put to rest the attractive myth that a heathy older person can be at lower risk from COVID-19 than younger people with co-morbidities.

To this end, the renowned American Centers for Disease Control (CDC) provide a simple guidance, listing two rubrics for risk: 1) older adults - even without co-morbidity and 2) those with underlying conditions - at any age. (see: https://www.cdc.gov/coronavirus/2019-ncov/need-extra-precautions/people-at-increased-risk.html; accessed 25 Sept., 2020). So too did the Canadian Geriatrics Society make similar recommendations using age 60 + (unrelated to the presence or absence of risk factors) as the number at which risk begins to rise (see: https://cgjonline.ca/index.php/cgj/article/view/443/507; accessed 25 Sept., 2020).

Patients in nursing homes have been and likely will continue to be a particularly hard hit group, as has been observed in Europe (https://www.theguardian.com/world/2020/apr/13/half-of-coronavirus-deaths-happen-in-care-homes-data-from-eu-suggests), Canada and in the US (https://www.nytimes.com/2020/04/17/us/coronavirus-nursing-homes.html). In Israel, although the absolute numbers remain low relative to many other countries, institutionalized residents still make up about one-third of the COVID-19 victims. This is of course bad news but fortunately we have not witnessed the terrible scenes of neglect observed abroad. Fortunately, early on in the pandemic, the MoH published and at least partially enforced comprehensive guidelines as to how to deal with this sector (https://govextra.gov.il/media/16435/elderly-care-covid19.pdf; accessed 25 Sept., 2020) with a special team dedicated to dealing with this situation.

### “Lockdown” policies: ageism or age-protective?

In Israel, despite some protest, chronological age has been considered as one of the first criteria for social isolation or “stay at home directives”, and ultimately considered the last to be released from lockdown. There are several reasons to support such a consideration as well as counter arguments. These are addressed now, followed by a possible solution which attempts to balance the main conflicting considerations. Such guidelines need to be scientifically valid, transparent, workable, and insofar as possible, fair so that most in society will be able and agree to buy into it.

#### Ageism

However before moving on, one must address the complex issue of “ageism”. It was the late, great gerontologist Dr. Robert Butler who first defined the term, which according to the WHO constitutes “ … the stereotyping, prejudice, and discrimination against people on the basis of their age [alone]. Ageism is widespread and an insidious practice which has harmful effects on the health of older adults. For older people, ageism is an everyday challenge. Overlooked for employment, restricted from social services and stereotyped in the media, ageism marginalizes and excludes older people in their communities.” (see: https://www.who.int/ageing/ageism/en/ accessed 25 Sept., 2020).

Clearly, the use of someone’s chronological age to stereotype and discriminate (in the social sense of the word) is completely unacceptable. For example, age cannot be a criterion for a job for which it is not relevant (e.g. accounting, childcare or academic promotions, etc.). But some well-accepted regulations do use chronological (not even “biological”) age as an inclusion and exclusion criterion for certain types of work. Common sense is called upon here. For example, it is unlikely that most passengers, even the most gerontophilic, would feel completely comfortable watching two otherwise healthy 90 year old pilots enter the cockpit to preside over a 12 h trans-Atlantic flight. In recognition of this logic, even though one could claim it is an expression of ageism, most authorities restrict commercial airline pilots’ license to those younger than 65. Even then they must also prove that they are healthy (https://www.easa.europa.eu/sites/default/files/dfu/EASA_REP_RESEA_2017_1.pdf).

In contrast, a situation in Israel where sadly ageism is still definitely at work can be found in the age-mandatory retirement laws governing academia and the civil service. In our view these policies are wrongheaded but this issue is beyond the purview of this paper.

#### Reverse ageism?

On a more positive note, chronological age *is* used to entitle a universal pension or rights for certain services (e.g. in Israel, homemaker hours according the Nursing Care Act, etc.). Age also confers discounts on public transport to wealthy older persons rather than poorer younger ones – a case of “reverse ageism?” More trivial perhaps, but still relevant to this discussion, there seems to be no serious societal objection to older person receiving discounts to films, concerts and even municipal taxes, simply on the basis of age alone. One could even argue that we use age to discriminate to a significant degree against younger people, e.g. forcing (almost) all Israeli youth to register for the military draft at age 17 and obligating most of them to serve their country and possibly endangering themselves to protect their elders for at least 2–3 and sometimes many more years during their own early and formative years, returning to serve in the reserves for many subsequent years. Might this not be considered another example of “reverse ageism”?

#### Grey on Grey ageism

Writing recently in the BMJ, one British authority pointed to a disturbing phenomenon. “What is undoubtedly ageist is a collective fear of ageing and death in our societal and media values, meaning that appearing old is seen as being diminished, invisible, and unvalued by society. This in turn leads to older people themselves ‘othering’ any older people they see as being vulnerable, different from their more youthful and active selves. This can lead to ‘grey on grey’ ageism.” (ref [10]). True, but even worse in our view has been society’s lack of preparedness for how this virus could affect older persons that constitutes the true expression of discrimination against older persons – both in Israel and abroad.

#### Autonomy versus beneficence (aka “paternalism”)

For those older persons who, despite knowing the facts, might chose to take risks and expose themselves to this virus, some will argue against an ageist “paternalism” that would prevent them from exercising their human rights. This means that a person at risk at any age must be free to make an individual decision as to whether he/she is willing to go back out into society, take the risk of falling ill with COVID-19 and accept the consequences, however dire they may be. This claim, in contrast to the one re the older airplane pilot falling ill and endangering all passengers, assumes that older persons are only endangering themselves were they end up needing hospitalization or an ICU bed.

Indeed, should this second wave respond to steps being taken and wane quickly and Israel be assured that we have enough ICU beds to manage any surge, this claim will be valid and the older person or anyone with other risk factors must be free to take their chances. However, as we are now riding a second wave, this argument (supporting autonomy) seems much less valid in that it will be also be crucial to protect the stock of hospital and ICU beds so that they would be available for as many as possible – young and old. In such a case it may well become necessary to be stricter in regulations taking distributive justice into account by enforcing isolation of all high risk groups, older persons among them.

For its part, the Israel Gerontology Association (long the lead in many important age advocacy initiatives) joining a coalition with four other organizations, have mobilized in the direction of protecting the autonomy of older persons against what they view as an ageist paternalism. They argue that using chronological age as a sole criterion discriminates against some older healthy persons who they claim may even be at less risk than younger sicker people. Recently this coalition responded to the Joint Questionnaire of Special Procedures Mandate of Older Persons sent out by the UN (see: https://www.ohchr.org/EN/HRBodies/SP/Pages/Joint-questionnaire-COVID-19.aspx; accessed 25 Sept., 2020).

The coalition did make some important points, warning for example of the dangers posed by ageism to Israel’s older population brought on by the COVID-19 crisis (personal communication Prof Yitzhak Brick). Unfortunately, the coalition members also made the incorrect claim that chronological age is not an independent risk factor for COVID-19 complications, despite the clear evidence that it is. For example, in error they offer that, “[F] irst, it was clear that there is no difference between old and young people with regard to infection. Secondly, most of the older persons who died from the disease suffered from co-morbidities and severe health risk factors. 40% of the people who died from the Covid-19, came from long term facilities.” Their statement goes on, claiming, “ … .that the chronological age cannot be the sole criterion [as to who needs to stay at home], *as some people at high age are fit and healthy and others who are younger can be sick and frail,* old persons are not all the same. Policy makers should not depend on the chronological age when they decide about who has the right to go out of his home or not, and the decision should be made by the person himself [italics ours].”

This error of fact, the claim that chronological age is *not* an independent risk factor, does no service to the elderly and will in fact mislead those who need to make difficult decisions in the weeks and months ahead. Others, such as the British Society of Gerontology have made similar claims (ref [11]). There is also a growing protest movement among some older people in Israel (ref [12, 13]) as well as in Europe (https://www.wsj.com/articles/older-europeans-reject-calls-to-remain-in-isolation-as-lockdowns-ease-11589112002; accessed 25 Sept., 2020) arguing against the justification of such strictures.

Beyond the issue of ageism, this argument rejects any offer of beneficence, adducing autonomy as the highest ethical value. However, most liberal democracies do put a limit on such considerations in other relevant domains and most citizens will accept these restrictions as reasonable. For example, at any age, one must wear a seat belt when driving. Societies demand such steps not only to protect the individual and his/her family (principle of beneficence, aka “paternalism”) but also in order to preserve the commons (principle of distributive justice). In the absence of such strictures, society would be more likely to lose the productive years left to an individual who is killed or badly injured in a car accident. In the spirit of both beneficence and distributive justice, lowering the costs to society of premature death or the subsequent rehabilitation of those who survive a car accident seems a reasonable consideration which justifies the (partial) curtailment of a citizen’s autonomy.

Closer to the elder pilot argument is the dire effect that too many people (at any age) simultaneously falling ill with COVID-19 would have on hospital services - particularly but not exclusively ICU/ventilator beds (principle of *distributive justice*). Although we are still not (yet) in that dire situation in Israel, this is not just a theoretical argument as we have seen examples from around the developed world of health services becoming overwhelmed or coming very close to doing so as a result of too sudden and heavy a surge on bed and personnel availability (ref [5, 14]).

According to this argument, it is not only in the personal interest of high risk people (older persons as well as younger people with co-morbidity) to make every effort to avoid infection. As well, the argument goes, they should do so in order to help maintain the viability of a health system given that this organization needs to be capable of looking after them should they (or their children or other younger persons) fall ill.

Of interest is the sense that despite the recent relaxation of formal strictures, preliminary data from Israel’s largest Health Fund (Clalit) suggest that older persons seem to be voting with their feet to protect themselves. They seem to be voluntarily observing stricter behaviours than those demanded by the Israeli government. As pointed out in *The Times of Israel* on 12 July, 2020, Though Israel’s infection rate has soared to some 1200–1400 new cases a day in recent days, the percentage of serious cases has been far lower. For example, at the height of the first wave in mid-April, some 180 of a total 9800 active cases were considered serious, or about 1.8%. On Saturday [11 July, 2020], 134 of 18,296 cases were considered serious, or about 0.7%. (see: https://www.timesofisrael.com/at-risk-groups-less-hard-hit-in-2nd-wave-causing-fewer-serious-cases-analysis/; accessed 25 Sept., 2020).

#### Older persons who advocate for the strengthening of distributive justice

Against the claim of “ageism” and in the spirit of supporting both “distributive justice” and intergenerational solidarity, others feel that especially in a situation of a critical imbalance between demand and ICU resources, it is indeed justified to use chronological age as a criterion (among others of course) for the allocation of scarce resources. For example, a noted American medical ethicist Franklin Miller (himself 71 years old) offered, “If demand for ventilators keeps growing and further outstrips supply, I believe it could be justifiable as a matter of policy to forgo mechanical ventilation for all patients 70 years of age and older who have a medical condition that puts them at elevated risk of death, such as chronic renal disease, cardiovascular disease, diabetes, and chronic lung disease” (ref [15]). Another authority, Larry Churchill went even further offering (“as I approach my 75 year”) his own personal ethical approach which would give priority to a younger person (ref [16]). Closer to home, A.B. Yehoshua one of Israel’s foremost writers and thinkers, expressed himself in a similar vein (ref [17]).

Not all will accept nor support this stance but it does seem to be a position taken by at least some older persons. Of interest, colleagues from the field of social gerontology have objected that even such self-sacrifice is in their view still ageist. Obviously, none of the three abovementioned distinguished older persons would agree. Undoubtedly they would hold that not allowing one to take this approach constitutes an unjustified attack on their autonomy.

#### Possible need for ICU/ventilation triage (distributive justice)

Should the present second wave tower high enough to threaten to overwhelm Israel’s limited supply of ICU and ventilator stock (as was observed in Italy, Spain and NY State several months ago), the need for difficult choices will inevitably arise. Much has been written about the vexed subject of ventilator triage (ref [18–20]). Relevant statements have also been published by the Israel Geriatrics Society in Hebrew on the website of the Israel medical Association (ref [21]) and in a modified English version in a geriatrics journal (ref [22]) as well as by a public commission set up by the MoH (ref [23]).

Should they wish, and we believe many might elect to do so, a significant number of older persons could voluntarily avoid ending up a triage case or at least ensure clarity relating to their wishes should they reach such a fork in the road. In a thoughtful piece in the NEJM, Aronson recently offered, “I know many happy engaged elders in their 70s, 80s, 90s, and 100s … who would not want to be put on a respirator … Patients and [the US] health care system would be better served if all adults and elders use some of the spare time created by our new, home–confined lives to discuss and document their care preferences, whether the goal is aggressive, supportive or palliative care.” (ref [24]).

Unfortunately, Israel is still quite far behind other industrialized countries in this domain, only recently beginning any discussions on the possibility of “a good death” (ref [25]). Even worse, it is still very difficult and expensive to legally appoint someone an enduring power of attorney which is another way to reduce conflict and misunderstandings over this fraught issue. Furthermore, problems of cognitive decline, impaired vision and hearing, not to speak of linguistic mismatch between health care personnel and their older patients (not uncommon in Israel), could interfere with an older persons’ understanding their situation and expressing their relevant wishes.

Aronson bemoans the dire effects of the absence of such planning (for any reason) which “ … increases the suffering at the end of life …” with the presence of such documents helping “ … people with serious or life-limiting illness to live and die according to their personal preferences” (ref [24]). Relevant efforts must swiftly be made to avoid the maleficence that might follow from ignoring this urgent need.

#### Ensuring non-maleficence (primum non nocere)

For various reasons related to history and culture, Israeli elders, even those with a very short life span (including people with advanced cancer or severe dementia) are often subjected to far more aggressive treatment than would be the case in other western countries. Sadly, this phenomenon is observed even when such interventions are clearly futile and painful (ref [26, 27]). As such, in many cases, when an older person falls acutely ill in Israel, he/she may be subjected to invasive procedures and an admission to ICU etc. This despite the fact that had the older person really understood what was actually involved, they may well not have agreed to such an intervention. It is thus society’s solemn duty to ensure that older persons clearly understand what the automatic fallback options are should they not have made their prior wishes known.

Furthermore, as addressed above, it is essential that older persons understand the fact that age is an independent risk factor for COVID-19 complications and death. Suggesting otherwise, despite the clear evidence that it is, does them a terrible disservice in that they may act to endanger themselves by thinking that as a “healthy” older person they are at lower risk than they actually are.

### How to manage this second wave?

As Israel enters its second lock-down (in late September coinciding with the Day of Atonement [Yom Kippur]) It is worth studying the approach by Pueyo alluded to above (ref https://medium.com/@tomaspueyo/coronavirus-the-hammer-and-the-dance-be9337092b56). But the truth is that at this stage, no-one has yet choreographed either a perfect “hammer” or “dance”. All agree that it is economically and socially unsustainable to keep most of the population locked down and laid off indefinitely, even at the cost of more COVID-19 deaths. Without health, there is no wealth; but the opposite is also true. As such we must open up our societies as quickly but as we safely can.

All of the suggestions offered below, in order to be humane (encouraging beneficence and maximizing non-maleficence) and fair (distributive justice) and in part to compensate older citizens for having to wait until the younger ones are first “released”, will require that society take some important steps in parallel. These would include ensuring that during the hammer and even for some time afterwards, older housebound persons would have their daily needs met – material, medical and psychological. Space does not allow us to go into detail but an example would be special “older hours” for food stores, facilitated home delivery, availability of handymen, plumbers and electricians who would be on call via a central number, etc. and other relevant supports.

All of these suggestions require that the MoH, along with other relevant government agencies, keeps its finger on the pulse of the epidemic – opening the faucet, testing and opening or closing it further depending on the results of extensive and focused testing.

### A step-by-step proposal

1. With respect to the at-risk populations (those with relevant medical illnesses *and* older age), so far, even though the number of infected person is rising once again, at the date of writing (late October, 2020) the program recommended herein is still voluntary. This however could change should the situation worsen significantly. One hopes that relevant professional organizations (such as the Ministry of Health and the Israel Geriatrics Society) NGOs such as JOINT-ESHEL and lay bodies will use their influence to convince older people and others at high risk to voluntarily follow these guidelines, as they are both in their own individual interest and that of society’s – with or in the absence of a lockdown. Many of these steps have been taken previously but we are aiming at a rapidly moving target. However, should we reach a catastrophic situation of overwhelmed emergency rooms, insufficient ventilators (and/or the team members needed to manage them), mandatory lockdown of all high risk persons of any age might be required.
2. Save lives and protect the system (in that order). Although it should be obvious by now, the three essential steps are physical distancing, wearing a face mask and frequent hand washing. And it is indeed distressing to observe how few young people in Israel (and others around the world) seem to have internalized the need for such simple but efficacious behaviours. There are even a few world leaders who demonstrably refuse to cover their offending upper airways although fortunately this is largely not the case in Israel. To this end, the state needs to more aggressively ensure the promulgation, explication and enforcement of relevant regulations and the supply of appropriate kit in public spaces. Here we would expect the MoH to lead the way with public health announcements supported by the media, neither of which have as yet excelled in this domain.
3. It is critically important that public personalities, cultural figures, athletes and above all politicians follow and are seen to follow the rules. It is particularly difficult to ask the populace to act in a compliant manner especially when at least three of Israel’s leaders (all over age 70), Prime Minister Benjamin Netanyahu, President Ruby Rivlin, and most egregious of all, the then Minister of Health (!) Rabbi Yacov Litzman all shamelessly broke the MoH rules over the recent Passover holiday. And in early July the newly appointed Minister of Health Yuli Edelstein also flouted his own ministry’s regulations. We are not alone, as many leaders from around the world have acted in a similar irresponsible way, but in this domain all politics are local. Of interest is the welcome public apology recently offered by Pres Rivlin for his un-leader-like behaviour during the last major Jewish/national holiday of Passover (see: https://www.timesofisrael.com/as-lockdown-set-to-begin-rivlin-apologizes-for-leaders-virus-failures/#gs.gk20fz accessed 19 sept,2020). However, to the best of our knowledge, he is the only miscreant who has offered any such contrition.
4. All of the steps outlined herein are mutually supportive. Sensible physical distancing must be explained and defined so that older persons aren’t unnecessary “imprisoned” in their apartments – in other words, needlessly distanced *socially*. For example there is very little risk involved in meeting children and grandchildren outside in a park or garden strictly separated by 2 m and wearing masks, etc. Overly stringent, contradictory and irrational guidelines offered by the MoH characterized the first wave and caused significant and entirely unnecessary suffering among older persons, especially but not only in Sheltered Housing (*diur mugan*).
5. In the fall and early winter it will also be especially important to ensure a robust influenza vaccination program with wide availability of anti-flu medications (e.g. oseltamivir [*Tamiflu*]) given that the rise in flu cases which usually begins in November will be superimposed on the ongoing COVID-19 pandemic. In this vein health personnel must be encouraged and perhaps even legislated to take a mandatory flu vaccine, given the disappointingly low rates of uptake by this crucial sector in Israel in past years. Further clinical guidance must be offered to older persons and physicians in the field as to how to handle a patient presenting with non-specific “flu-like” symptoms from Nov-March – swabbing, an algorithmic strategy if positive or negative for flu or SARS-CoV-2, etc.
6. All persons over age 60, even without co-morbidity, must clearly understand that they are at increased risk for complications and death should they become infected; the older, the greater the danger. This is the case even for an otherwise robust older person. Comorbidity adds risk to chronological age; patients and their doctors must understand this clearly. Despite pushback by some ill-informed Pollyanna’s, this message must be forceful and clear. Here NGOs such as the Israel Association of Gerontology and JOINT-ESHEL could help spread the evidence-based word.
7. Many who fall seriously ill may elect to be hospitalized and if necessary ventilated (see above). However, there will be those who do not wish to undergo this procedure, instead opting for a more palliative approach. In order to exercise their autonomy, all adult citizens (especially those with any relevant co-morbidity and all those > 60 years) should sign an advance directive. These are available on line from the MoH (see https://www.health.gov.il/Services/Citizen_Services/DyingPatientLaw/Pages/DyingPatientRequest.aspx). As well, it is advisable to prepare an enduring power of attorney (ייפוי כוח). It is most unfortunate that in Israel this process is so complex and expensive and the Ministry of Law shares responsibility for this dire situation. Hopefully in the near future, it will be simplified and further encouraged.
  In the true interest of their older clients, relevant groups such as the Israel Association of Gerontology, JOINT-ESHEL and other members of the abovementioned “Coalition” should lobby to simplify these procedures and to convince more citizens to take this essential step in order to protect the exercise of their autonomy.
  In the absence of such guidelines, faced with a patient ill with COVID-19, clinicians will find it difficult to know what the individual patients’ wishes are re ventilation. From the legal point of view in Israel, family members have no formal say in such decisions unless they are the legal guardian (אפוטרופוס) of an older person or have the enduring power of attorney mentioned above.
8. At present (late October), while under increasing strain, Israel’s hospitals are still just able to cope with the influx of COVID-19 patients. However, this balance could change rapidly and should a severe mismatch between needs and resources develop, one would seriously have to consider the need for triage (see above). In this domain much has been written about chronological age *alone* not being a relevant consideration but most understand that this factor cannot be ignored. Furthermore, adding a moral twist to the debate, doing so may be considered by some as practicing ageism. However, in our view, while age *alone* should not be used as a factor in triage decision making, common sense and the fact that mortality goes up logarithmically with age as well as the chance of coming off a ventilator becomes vanishingly small, it cannot be ignored. The MoH would do well to introduce these guidelines into the legal regulations where relevant.
9. Those persons with significant comorbidity (at any age) are considered as belonging to the older person (60+) category. Under present conditions they should be advised, insofar as is possible, to stay "shielded". However, using similar logic to that pertaining to ventilator triage, should the situation worsen significantly, in the spirit of maximizing distributive justice, consideration would be given to enforcing such behaviour.
10. Returning to *all* older persons (60+), depending on the results of the steps described above, if the situation once again allows, they should be encouraged to return to normal function - but only gradually and carefully. This would pertain to all “vulnerable” persons at any age with serious underlying health conditions (as previously outlined) and those whose immune system is compromised such as by chemotherapy for cancer and other conditions requiring such therapy.
11. Unfortunately much poor (and confusing) advice was disseminated to older persons during the initial lockdown with the MoH failing to provide timely and accurate advice re prevention and health promotion. As we have now entered a new lockdown, the following recommendations would pertain. Even now these guidelines are relevant to all older persons and any younger people at high risk.

i. Although not always easy to do so accurately, each person can try to determine his/her own risk from the coronavirus and make decisions accordingly. The MoH should help by providing simple evidence-based guidance to people, taking into account one’s risk profile, medical history and, if necessary, consultation with the individual’s family doctor. The MoH has published a schema on their website, but it is difficult to find, confusing and not known to most older persons.
ii. Even in the event of a strict “lockdown”, persons of any age should still get out of their apartment and enjoy as much physical exercise as possible. There is no good medical rationale to prevent people at any risk not to walk, jog, outdoor yoga /tai chi, etc. - as long as masking and physical distancing are maintained.
iii. Re essentials (health, shopping, essential services), people at risk should get as much help as possible from delivery services, friends, family and the local authorities in order to minimize going out for these needs. Some people will need assistance from the state/municipality to manage. Examples of sensible social engineering taken in other countries include having supermarkets maintain certain hours for high risk persons and directing shoppers through aisles in a “one way” direction while also enforcing the two meter rule. To the best of our knowledge, none of this exists today The Health Funds (*kupot haHolim*) will need to be ready to provide adequate medical services at home and/or at specially configured clinics at specific hours in the day.
iv. All persons at risk need to maintain strict physical (not social) distancing including from family members (especially those aged 10+). With any outside contact, masks must be worn by all and no physical contact is allowed, including for example, passing plates of food back and forth. Family visits outside in a private garden or public park should be allowed as long as everyone stays more than 2 m apart.
v. Some older people may choose, in the spirit of maximalising distributive justice and out of a sense social solidarity towards the younger generation (as for example expressed by A. B Yehoshua among others alluded to above ref [15–17]) and out of concern regarding their individual risk, to maintain even stricter social isolation as well as to give priority to younger persons. This decision should be neither minimized nor mocked. Whatever one’s thoughts about ageism, this choice is to be honoured and respected as a legitimate expression of the at-risk person’s autonomy. We live in a society where certain younger age and occupational groups sacrifice for the health, safety and security of those older than them and this must be a bi-directional phenomenon – especially between consenting adults.
vi. As addressed above, all older persons should be encouraged and if necessary helped to make and document decisions about advanced directives so that their wishes can be respected should their health suddenly deteriorate. This expression of autonomy is of paramount importance, especially in times of crisis and uncertainty and given the default option of aggressive ICU and ventilation measures too often taken in this country. Should a triage situation develop,clarification of this domain will also help reduce family uncertainty as well as decreasing unnecessary pressures on the health care system.

12) As alluded to in sidebar 1, Israel enjoys a population of approximately 1.1 million citizens over 65 years, 97.6% of whom live in the community. All of these recommendations refer primarily to older persons dwelling in the community However, they would not be as applicable to the 22% of older persons receiving homemaker care according to the Nursing Law (חוק סיעוד) or who had an authorization for a personal attendant (usually a foreign worker). Such people will be much frailer than the usual older person, exhibiting even a higher prevalence of co-morbidity and cognitive decline. Another vulnerable sector would also not be included in this schema, that is persons in institutions for older persons (approximately 23,600, that is 2.6% of the elderly) with the possible exception of those more independent elders living in sheltered housing (דיור מוגן). As mentioned above, the MoH has designed an ongoing mechanism (*Magen Avot*) meant to protect this extremely vulnerable population. Among other things, this program ensures an adequate supply of PPE as well frequent as PCR testing of both residents and staff. After a rocky start this program now seems to be working quite well and offers area example of what Israel got right during the pandemic (ref [28]).
13) Planning needs to consider a sensible exit strategy from the ongoing second wave. These recommendations should be instituted gradually: releasing first those 60–69 years old; then 70–84 and finally all 85+. Although such age categories are admittedly arbitrary, they clearly represent the increasing risk of the average person in each group from COVID-19 (less clinical reserve, higher likelihood of co-morbidity and shorter life expectancy, etc.) as one climbs the age scale.
14) Outreach is needed to populations which traditionally have less access and/or trust in the healthcare system such as citizens from Arab and Ultra-Orthodox communities where infection rates are increasing more than in the general population. This can be done by having citizens from those communities actively involved in the decision making process and encouraging local leadership to take an active role in disease prevention and management.

### Future directions

In this paper we have tried to address the vexed issue of age; how a society such as Israel’s should make every attempt to meet the needs of older persons during the pandemic while taking into account those of the wider society as well as the sometimes conflicting principles of medical ethics. Space does not allow us to deal with all relevant issues and for some we can only outline the topic. Please see sidebar 4.

### A final (personal) word from the older author (AMC)

Even today and especially as we ride and try to balance on the second wave without plunging into the roiling seas, this proposal puts much onus on Israel’s senior citizens, many of whom have not had an easy life. This will be the case whether or not these guidelines are statutory or voluntary. Just as they may have begun to enjoy retirement, hobbies, their grandchildren etc., older persons are once again to be restricted (at least partially) by this terrible pandemic. It must however be kept in mind that it is the virus, not society which is responsible. This proposal asks a heavy price of older persons, i.e. to wait inside and struggle relatively alone for longer than younger people. But it is logical and meets the criterion of soft utilitarianism alluded to above (the greatest good to the greatest number.)

I am almost 71 and, according to this proposal, will have to wait my turn until I am allowed and/or allow myself more freedom - perhaps for quite a while. As are the two older medical ethicists and A.B. Yehoshua quoted above (ref [15–17]), I am willing to do so because I believe in the science, logic and moral approach of this “dance”. In addition, in social solidarity with younger people, I am willing to take these steps for the sake of my children and their generation which is the one which will drive the economic, defense and social engines of our society out of this crisis. And in the end, as an older Israeli, I (and I know of others) am willing to do this for the sake of our society.

## Conclusion

While many infected persons are asymptomatic and most survive the SARS-CoV-2 virus, COVID-19 can be a serious disease, especially for those with co-morbidity and for many older persons, even without. The SARS-CoV-2 virus has caused illness and death and wrought severe socio-economic disruption for people at all ages across the globe (see https://www.economist.com/international/2020/09/26/the-pandemic-is-plunging-millions-back-into-extreme-poverty). Given the iron laws of biology, on average healthy older persons are at higher risk than younger, even unhealthy people. As such, society needs to protect all of those particularly susceptible to this virus – both from the disease as well as from the ill effects of the necessary constraints on their freedoms necessitated by this worldwide emergency. But equally important, governments must act transparently and solely in the interests of citizens. Finally, they need to ensure a fair distribution of resources - especially if society is faced with an acute shortage. The trick will be in getting the balance right.

## Supplementary information

**Additional file 1.** Sidebar 1: The new virus in Israel: a young-old country.

**Additional file 2 **Sidebar 2: The Approach of Formal Jewish Law (*Halacha*).

**Additional file 3.** Sidebar 3: Biological ageing: what is it?.

**Additional file 4.** Sidebar 4 Future directions.

## Data Availability

not relevant.
