# Supplementary material for: Age, ageing, ageism and “age-itation” in the Age of COVID-19: rights and obligations relating to older persons in Israel as observed through the lens of medical ethics
Source: Isr J Health Policy Res. 2020 Nov 12;9:64. doi: 10.1186/s13584-020-00416-y (PMC7658431; doi:10.1186/s13584-020-00416-y)
Supplement: Supplementary file 1 — Additional file 1. Sidebar 1: The new virus in Israel: a young-old country. [file 13584_2020_416_MOESM1_ESM.docx]

**Sidebar 1: The new virus in Israel: a young-old country**

Compared with other developed countries, Israel's population is still young in years, with relatively high birth rates and a lower proportion of older persons (65 years +) who make up just over 11.5 % of her population. Still, the absolute number of older people in the country is growing rapidly (ref 1-1). Life expectancies at every age are among the highest in the world, with increasing numbers of people living into very old age (85 + years).

Israel has a top notch, highly socialized national health system (ref 1-2) offering mostly free care from cradle to grave. Community and institutional care are both widely available, but of particular relevance to the COVID-19 pandemic, Israel institutionalizes a lower percentage of her older persons than do many other developed countries at 23.6 persons 65+ per 1000 population (OECD average 47.2; lowest Greece at 4.5; highest Luxemburg at 82.8) (ref 1-3). In Israel, a large proportion of frail older persons who in other countries might otherwise have been institutionalized, are cared for at home often with the help of a legal foreign worker. This shielding practice of keeping such a high proportion of Israel's frail older persons cosseted in their own homes may in part explain her (still) relatively low death rates from COVID-19.

**References for sidebar 1**

- 1. Dwolatzky T, Brodsky J, Azaiza F et al. Coming of age: health care challenges of an ageing population in Israel. Lancet 2017; 389 (10088): 2542-2550.
  2. Clarfield A. Mark, Manor O, Bin Nun G, Shvarts S, Azzam ZS, Afek A, Basis F, Israeli A. Health and health care in Israel: an introduction. Lancet 2017; 389:2503-13. <https://doi.org/10.1016/S0140-6736(17)30636-0>
  3. OECD: Health at a Glance 2019.OECD Indicators. OECD Publishing, Paris fig 11.26, p 213; doi.org/10.1787/4dd50c09-en)
