# Supplementary material for: Age, ageing, ageism and “age-itation” in the Age of COVID-19: rights and obligations relating to older persons in Israel as observed through the lens of medical ethics
Source: Isr J Health Policy Res. 2020 Nov 12;9:64. doi: 10.1186/s13584-020-00416-y (PMC7658431; doi:10.1186/s13584-020-00416-y)
Supplement: Supplementary file 2 — Additional file 2 Sidebar 2: The Approach of Formal Jewish Law (Halacha). [file 13584_2020_416_MOESM2_ESM.docx]

**Sidebar 2: The Approach of Formal Jewish Law (*Halacha*)**

For its part, although oddly enough not much discussed publically, formal Jewish law would very strongly support the approach offered herein. As opposed to the usual perspective expressed in secular ethics, *Halacha* puts as much or even more emphasis on an individual's obligations to his fellow citizens and to society than to his/her rights.

Autonomy, which is based on the approach espoused by universal human rights, is not a supreme value in Jewish medical ethics. For example, one of the founders of this discipline, Rabbi Immanuel Jakobovits wrote: "…in Judaism we know of no intrinsic rights. Indeed there is no word for rights in the very language of the Hebrew Bible and of the classic sources of Jewish law. In the moral vocabulary of the Jewish discipline of life we speak of human duties, not of human rights, of obligations not entitlement. The Decalogue is a list of Ten Commandments not a bill of Human Rights.

"In the charity legislation of the Bible, for instance, it is the rich man who is commanded to support the poor, not the poor man who has the right to demand support from the rich. In Jewish law a doctor is obligated to come to the rescue of his stricken fellow-man and to perform any operation he considers essential for the life of the patient, even if the patient refuses his consent [beneficence trumps autonomy] or prefers to die. Once again, the emphasis is on the physician’s responsibility to heal, to offer service, more than on the patient's right to be treated. " (ref 2-1)

The late medical ethicist, Benjamin Freedman has pointed out that what distinguishes a Jewish approach to moral dilemmas is an emphasis on a "duty-based" ethic as opposed to a "secular" ethic based on rights (ref 2-2). For his part, another authority Cover concurs explaining that, "[E]very legal culture has its fundamental words.… The word 'rights' is a highly evocative one for those of us who have grown up in the post-enlightenment secular society of the West.... Judaism is, itself, a legal culture of great antiquity.... When I am asked to reflect upon Judaism and human rights, therefore, the first thought that comes to mind is that the categories are wrong. I do not mean, of course, that basic ideas of human dignity and worth are not powerfully expressed in the Jewish legal and literary traditions. Rather, I mean that because it is a legal tradition, Judaism has its own categories for expressing through law the worth and dignity of each human being....The principal word in Jewish law, which occupies a place equivalent in evocative force to the American legal system’s 'rights', is the word 'mitzvah' which literally means commandment but has a general meaning closer to 'incumbent obligation ' ” (ref 2-3).

Thus from a Jewish perspective there would be no ethical problem with the government enacting laws to protect individuals and/or restricting their individual rights for the benefit of society. For example, many rabbis forbid smoking. Halacha also recognizes the power that government has in these matters and would not object to a law that limits the movements of older people in order to protect them from the virus.

In addition, the concept of *Pikuach Nefesh* (saving a life) is one of the highest values in Judaism and all the commandments in the Torah (except murder, idolatry and adultery) are deemed expendable in the face of danger to life. Thus from a Jewish religious perspective maximum efforts should be expended in order to potentially save lives and this would pertain to the pandemic as well.

Oddly however, despite the presence in the Knesset [parliament] governing coalition of two Haredi (ultra-Orthodox) political parties, these issues have not been a part of the discussion in any constructive way.

**References for sidebar 2**

2-1 Jakobovits I. The Timely and the Timeless: Jews, Judaism and Society in a Storm-tossed Decade. London: Vallentine Mitchell Publishers, 1977, p 128

2-2 Freedman B. Duty and healing: foundations of a Jewish Bioethic. New York: Routledge, 1999).

Cover R. Obligation - A Jewish jurisprudence of the social order. Journal of Law and Religion 1987: 5: 65–74
