# Supplementary material for: Age, ageing, ageism and “age-itation” in the Age of COVID-19: rights and obligations relating to older persons in Israel as observed through the lens of medical ethics
Source: Isr J Health Policy Res. 2020 Nov 12;9:64. doi: 10.1186/s13584-020-00416-y (PMC7658431; doi:10.1186/s13584-020-00416-y)
Supplement: Supplementary file 3 — Additional file 3. Sidebar 3: Biological ageing: what is it?. [file 13584_2020_416_MOESM3_ESM.docx]

**Sidebar 3: Biological ageing: what is it?**

While it is indisputable that chronological age is also a risk factor for many (but not all) diseases, some argue that it is not age per se, but rather the "time exposed" (e.g. years of smoking) or "amount of exposure" (number of cigarettes smoked) which determines the chance of someone falling ill with lung or heart disease when they get older. That may indeed be the case, at least in part, although it does seem absurd to argue against the obvious, that increasing age itself at any stage, whether one is heathy or ill, is a strong predictor of death. One may even posit that many very old people actually die "healthy" with no evidence of any particular disease as a cause of death; but still, no one lives forever and in the end, ageing will kill us all, even if a specific disease doesn't. Why?

Put simply, mammalian ageing is the manifestation of the very gradual failure of an exquisitely efficient system of coordinated cellular (mainly DNA) self-repair. However, over time, due to a gradual accumulation of small errors in this robust mechanism, the genetic blueprint begins to transmit faulty instructions (ref 3-1). As these errors mount up, cells die and/or malfunction, organs fail; eventually "the centre cannot hold" and life ends. However, since H. sapiens has so much built-in biological reserve, this process takes many years and the gradual process of "ageing" is what we observe until the final collapse (aka death).

In a different but hardly less fractious era, Abraham Lincoln put it very well, characterizing ageing as resulting from the cumulative effects of "the silent artillery of time." Clearly, healthy living, a good diet, inheriting a robust genetic endowment, staying active both physically and mentally and having the good fortune to live with enough resources in a politically healthy society will encourage healthy ageing (ref 3-2). But no matter what, the inevitable cannot be staved off. Death will come for us all.

**References for sidebar 3**

3-1) DiLoreto R, Murphy CT. The cell biology of aging. Molec Bio of the Cell 2015; 26:4524-4531 DOI:10.1091/mbc.E14-06-1084

3-2) Clarfield A. Mark. Healthy life expectancy is expanding. J Amer Ger Soc 2018; 66:200-1 doi:10.1111/jgs.15165
