# Supplementary material for: Age, ageing, ageism and “age-itation” in the Age of COVID-19: rights and obligations relating to older persons in Israel as observed through the lens of medical ethics
Source: Isr J Health Policy Res. 2020 Nov 12;9:64. doi: 10.1186/s13584-020-00416-y (PMC7658431; doi:10.1186/s13584-020-00416-y)
Supplement: Supplementary file 4 — Additional file 4. Sidebar 4 Future directions. [file 13584_2020_416_MOESM4_ESM.docx]

**Sidebar 4: Future directions**

***Is chronological age a permissible consideration in rationing?***

As discussed above, chronological age is clearly an independent risk factor for complications and death in COVID-19 but there are others. Why should we not simply treat age as we might the NYHA classification for congestive heart failure or a person's frailty level - both equally prognostic? That is because there is a social and ethical danger in using chronological age alone as a consideration or even as a "tie breaker" for triage considerations. We are aware of the concern by some that however justified scientifically, using age might encourage or allow a more nefarious use of "ageism". In the spirit of supporting non-maleficence we need to more fully explore this vexed question in order to calculate the right balance while not shying away from the uncomfortable facts.

***The complex relationship between age and other co-morbidities***

While the data clearly indicate that chronological age is itself an independent risk factor and co-morbidities (eg hypertension, ischemic heart disease, etc ) are additive to risk, it is still unclear what the interactions are between and among these factors. For example, is there a difference between the effects of successfully treated versus uncontrolled hypertension as well as to how and whether these differences might influence the poor prognostic effect of age? To the best of our knowledge, these interactions have yet not been reported.

***The ethics of mandatory injunctions***

Ethical principles are often correct each on their own. It is when they clash in the real world that the situation is less clear. However, as we have pointed out throughout this paper, until we begin to approach a situation in which hospitals are close to being overwhelmed by demands which they cannot meet, a proportion of our recommendations remain moot. The difficult questions are when and how to activate involuntary measures such as the closure of a city or neighbourhood. What if the science showed unequivocally that the best way to handle this pandemic would be to strictly quarantine any person at high risk – all of the aged and many of those with relevant co-morbidities ? Could a democratic society handle such steps and how could it mitigate the risk to the human rights of those forced to comply? Although the data are scarce, this draconian approach may have been used in China, not a country in which the government holds human rights as critical value. (See: <https://www.nytimes.com/2020/08/25/world/asia/china-xinjiang-covid.html?auth=login-email&login=email> accessed 25 Sept.,2020).

***ICU/ventilator beds and older persons***

The question of how to determine the fair distribution and use of ICU/ventilation resources (equipment and trained personnel) predates the current pandemic. And the question is even more fraught in a condition of acute scarcity as we may well see soon enough in Israel and other countries. Thus the issue as it pertains to the COVID-19 pandemic does not appear in a vacuum. Even in "routine" times, such resources are relatively rare and fixed with guidelines existing as to who can access them. However, even if sufficient, in the spirit of supporting non-maleficence, they should not be foisted onto those who do not wish them. Nor should they be used on those whom they are not likely to save but will more probably just prolong a "bad death" (distributive justice).

***Other issues***

Many other ethical aspects remain to be explored such as the administration of investigational drugs under or outside clinical trials (non- maleficence) or issues relating to vaccination for COVID-19, both a person's participation in trials and then prioritization of vaccine doses when available (distributive justice). What are the ethical considerations in a condition of shortage should older persons require two doses and other high risk groups only one? In addition, neither the relevant socio-cultural aspects (including social disparities) nor the measurement of quality of life measures have been addressed in this paper.
